# Preventing High Fat Diet-Induced Obesity and Related Hepatic Steatosis by Chlorin e6-Mediated Photodynamic Therapy

**DOI:** 10.3390/ph17060729

**Published:** 2024-06-05

**Authors:** Pallavi Gurung, Junmo Lim, Yong-Wan Kim

**Affiliations:** Dongsung Cancer Center, Dongsung Biopharmaceutical, Daegu 41061, Republic of Korea; gp20@ds-pharm.co.kr (P.G.); jm15@ds-pharm.co.kr (J.L.)

**Keywords:** chlorin e6, high-fat diet, beagle dogs, obesity, hepatic steatosis, ovariectomized

## Abstract

Obesity and its associated hepatic steatosis have become a global concern, posing numerous health hazards. Photodynamic therapy (PDT) is a unique approach that promotes anti-obesity by releasing intracellular fat. Chlorin e6 (Ce6)-PDT was tested for its anti-obesity properties in male ovariectomized (OVX) beagle dogs, as well as male C57BL/6 and Balb/c mice. The 12 OVX beagles were randomly assigned to one of four groups: high-fat diet (HFD) only, Ce6 only, Ce6 + 10 min of light-emitting diode light (LED) treatment, and Ce6 + 15 min of light treatment. We assessed several parameters, such as body weight, adipose tissue morphology, serum biochemistry, and body fat content analysis by computed tomography (CT) scan in HFD-fed beagle dogs. At the end of the study period, dogs that were treated for 35 days with Ce6 and exposed to LED irradiation (660 nm) either for 10 min (Ce6 + 10 min of light) or for 15 min (Ce6 + 15 min of light) had decreased body weight, including visceral and subcutaneous fats, lower aspartate transaminase (AST)/alanine transaminase (ALT) ratios, and a reduction in the area of individual adipocytes with a concomitant increase in the number of adipocytes. Furthermore, C57BL/6 male mice following an HFD diet were effectively treated by Ce6-PDT treatment through a reduction in weight gain and fat accumulation. Meanwhile, Ce6-PDT attenuated hepatocyte steatosis by decreasing the epididymal adipose tissue and balloon degeneration in hepatocytes in HFD-fed Balb/c mice. Taken together, our results support the idea that Ce6-PDT is a promising therapeutic strategy for the recovery of obesity and obesity-related hepatic steatosis.

## 1. Introduction

Obesity, or excess fat accumulation, has become a serious health problem for humans and animals [1]. Overeating and inactivity are regarded as the causes of increased fat storage and further weight gain [2,3]. Obesity involves changes in the body’s metabolism and hormone levels, in addition to the buildup of a large amount of adipose tissue [4]. The obese condition reduces life expectancy and increases the risk of several complications, such as orthopedic disorders, glucose dysregulation, hypertriglyceridemia, cardiorespiratory dysfunction, liver dysfunction, immunological disorders, and carcinoma. Dietary habits, physical inactivity, and genetics are important risk factors for obesity in both humans and animals like dogs [5,6,7]. Other components, such as age, sex, gonadal status, and hormones, also influence the development of obesity [8,9]. Important risk factors for obesity include hormonal alterations and the resulting decreased metabolic rate from the absence of sex hormones. Estrogen is known to affect the quantity of adipocytes and has recently been shown to limit lipogenesis [10,11]. In a previous mouse investigation, estrogen therapy was found to reduce body weight and lipid metabolism in OVX mice [12].

Obesity is a low-grade chronic inflammatory condition in a variety of tissues, such as adipose tissue, that increases the risk of developing metabolic disorders like non-alcoholic fatty liver disease (NAFLD) [13,14]. NAFLD can be diagnosed on imaging or histology with the sign of chronic liver disease such as hepatic steatosis, where aberrant fat deposition occurs in at least 5% of hepatocytes, and if the percentage exceeds, it might result in liver cirrhosis or failure [15,16]. It is well known that physical activity, along with calorie restriction, can lower the incidence of fatty liver disease, but it cannot cure existing NAFLD [17]. Obesity-mediated NAFLD risk is associated with higher insulin resistance (IR) and inflammation. Obesity stimulates tumor necrosis factor-α (TNF-α)-associated inflammation, leading to IR. A prior study found that acute inflammation enhances adipogenesis in response to HFD, but inhibiting acute inflammation reduces adipogenesis [18,19]. In addition, sterol regulatory element binding protein 1c (SREBP-1c) regulates lipogenesis, and its activation in pathological settings can cause lipid dysfunction, contributing to disorders such as NAFLD by increasing the rate of fatty acid, cholesterol, and triglyceride synthesis. Signaling mechanisms, such as the phosphatidylinositol 3-kinase (PI3K)–protein kinase B (PKB, AKT)–mammalian target of rapamycin (mTOR) pathway, regulate SREBP activity and vary depending on physiologic conditions [20]. NAFLD frequently results in hepatic steatosis, which can be exacerbated by insulin resistance (IR), resulting in hepatic de novo lipogenesis (DNL) and reduced fatty acid transport. Other contributing variables include endoplasmic reticulum (ER) stress, autophagy disruption, mitochondrial failure, hepatocellular apoptosis, and an increase in inflammatory responses [21,22]. Previous research has linked severe steatohepatitis and fibrosis to elevated levels of TNF-α. TNF-α and interleukin-6 (IL-6) inhibit insulin receptor substrate activation, contributing to insulin resistance (IR) [23]. Oxidative stress has also been identified as the primary trigger for the advancement of steatosis to steatohepatitis, as well as a significant hallmark of non-alcoholic steatohepatitis (NASH) [24]. ER stress can produce hepatic steatosis by activating lipogenic pathways that further activate lipogenic genes [25]. However, the exact pathophysiology of NAFLD-induced steatosis is unknown, and no effective treatment has been identified in clinical settings [26].

Along with diet and exercise, pharmacological therapy is a conventional approach to treating obesity [27]. Dietary supplements, such as amphetamines, sibutramine, or orlistat, are used to reduce excessive food consumption; however, it has been demonstrated that most of these treatments have negative side effects or become ineffective with time, necessitating higher dosages [28,29]. As a result, more focused, effective, and efficient drugs and treatments are still required to treat obesity and its related illnesses. Here, we focus on a unique approach called photodynamic therapy (PDT), which is a safe, non-invasive treatment that combines light, molecular oxygen, and a photosensitizer (PS) [30,31]. It has already been documented that local PDT, mediated by verteporfin or indocyanine green, can reduce fat [31]. The major mechanism is that, after light exposure, PS causes adipocytes to die by producing singlet oxygen (1O2) and other reactive oxygen species (ROS) [29,32]. PDT momentarily disrupts the cell membrane of fat cells in adipocytes and releases intracellular fat through a mix of chemical and thermal processes. But the pores shut, and the cell membrane reverts to its original state when the laser radiation is turned off, sparing the fat cell. Moreover, adipose browning induction, which changes white adipose tissue into thermogenic adipose tissue, might be an additional pathway [31,33]. Though PDT often targets shallow fats, deeper targets such as adipose tissue and hair follicles can be targeted with intralesional PDT [34].

In our previous studies, we elucidated that Ce6-PDT can reduce HFD-induced obesity in mice. However, in this investigation, we seek to determine whether the effects of Ce6 followed by irradiation with a 660 nm light were successful in both HFD-induced OVX female dogs and male mice. Furthermore, we were also interested in studying the effects of Ce6-PDT on HFD-induced hepatic steatosis in a mouse model.

## 2. Results

### 2.1. Ce6-PDT Decreased HFD-Induced Obesity in OVX Female Beagle Dogs

**Figure 1:**
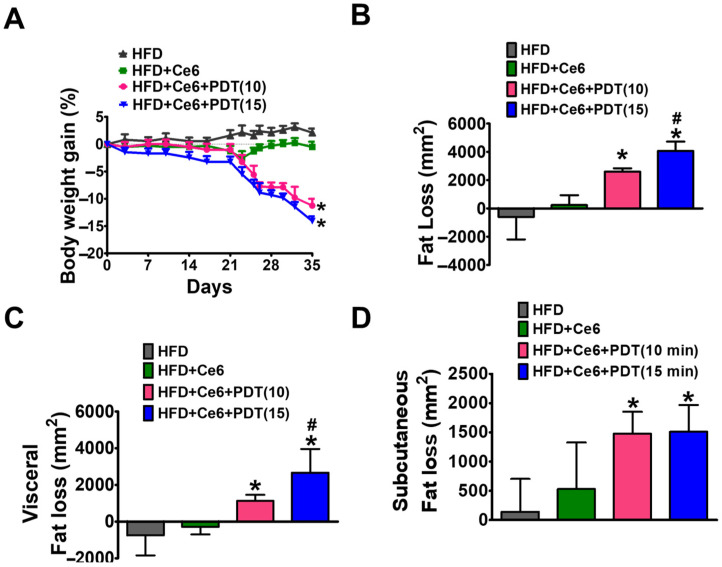
Ce6-PDT reduced body weight and fat in HFD-induced OVX female beagle dogs. After 49 days of regular HFD feeding, a 10% increase in the body weight of beagle dogs occurred (**A**) Body weight gain images of mice fat distribution in 0 and 35 days of drug treatment. (**B**) Fat loss. (**C**) Visceral fat loss. (**D**) Subcutaneous fat loss in all groups. The data are presented as the mean ± SEM (*n* = 3). * *p* < 0.05 compared with the HFD-only treated group. ^#^ *p* < 0.05 compared with the Ce6-only treated group (*n* = 3).

Next, to confirm the anti-obesity effects of Ce6-PDT, we induced obesity in female OVX beagle dogs by continuously feeding them a HFD. After 49 days of continuous administration of HFD, mice dramatically gained 10% body weight. On 49th day, Ce6 (2.5 mg/kg) with PDT (10 or 15 min) were treated to obese dogs up to 35 days (up to 84th day). Ce6-PDT significantly decreased body weight in both the groups in comparison to HFD-fed dogs (Figure 1A). However, a significant reduction in body weight was observed in the Ce6-PDT groups where LED lights were exposed for 15 min compared to when they are exposed to light for 10 min. A CT scan (S2) showed that HFD markedly increased the body weight of beagles and fat mass, especially the fat distribution in visceral and subcutaneous adipose tissue. Moreover, Ce6 with 15 min light treatments also resulted in significant fat and visceral fat loss in comparison to Ce6 with 10 min light treatment groups, whereas subcutaneous fat loss was similar in both light treatment groups (Figure 1B,C). The result indicates that prolonged LED light treatments have better anti-obesity effects on beagle dogs.

### 2.2. Ce6-PDT Ameliorated the AST/ALT Ratio and Blood Lipid Levels in HFD-Induced OVX Female Beagle Dogs

**Figure 2:**
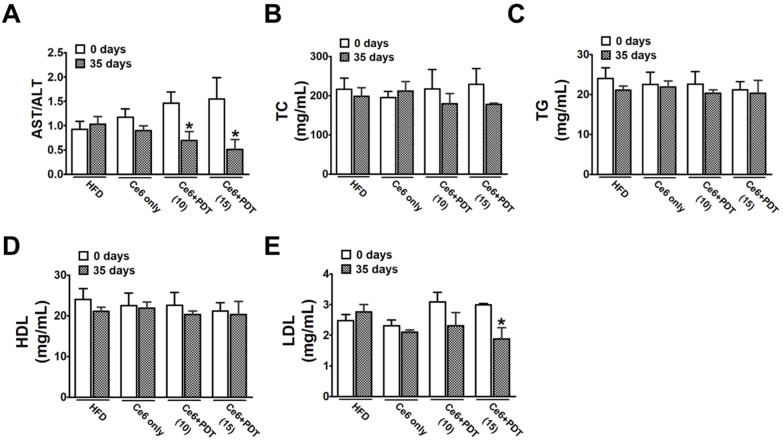
Changes in serum AST/ALT, TC, TG, HDL, and LDL between 0 and 35 days of Ce6-PDT treatment in OVX beagle dogs. (**A**) AST/ALT, (**B**) TC, (**C**) TG, (**D**) high-density lipoprotein (HDL) cholesterol, and (**E**) low-density lipoprotein (LDL) cholesterol. The data are presented as the mean ± SEM (*n* = 3). * *p* < 0.05 compared with the HFD-only treated group.

Hematological analysis showed that the HFD also increased the plasma AST/ALT ratio and lipid content, such as total cholesterol (TC) and triglycerides (TG), while decreasing the low-density lipoprotein (LDL) content. These findings show that HFD feeding successfully induces obesity in OVX beagles. Figure 2A–D show that, in the serum of the dog, the levels of AST/ALT, TC, TG, and high-density lipoprotein (HDL) were highest in the HFD, Ce6-only, and light-only groups. The Ce6 + 10 min-light and Ce6 + 15 min-light treatments both showed the lowest serum levels of AST/ALT, TC, and TG in comparison to untreated HFD-fed dogs. Groups exposed to Ce6 + PDT (10 min) and Ce6 + PDT (15 min) showed lower LDL levels than untreated HFD-fed dogs. However, the level of HDL was not affected by Ce6-PDT-treatment in HFD-fed beagles (Figure 2E). This indicates that Ce6-PDT has the capacity to remove liver damage and prevent lipid accumulation.

### 2.3. Histopathological Examinations of Adipose Tissues in HFD-Induced OVX Female Beagle Dogs

**Figure 3:**
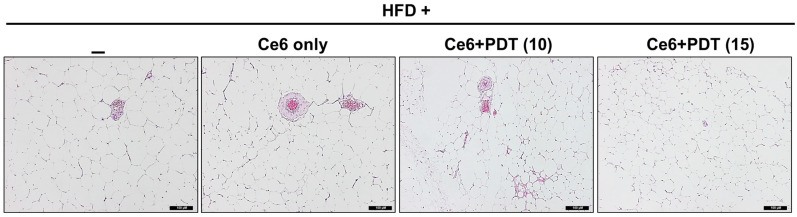
The representative histological appearance of the adipose tissue taken from HFD-fed and Ce6-PDT-treated OVX female beagle dogs from the abdominal wall-deposited fat pads. Magnification 100×; HE staining of high-fat diet (HFD)-fed group; Ce6-only treated group; Ce6 + PDT (LED-light exposed for 10 min)-treated group; and Ce6 + PDT (LED-light exposed for 15 min)-treated group (*n* = 3). Scale bar, 100 μM.

As shown in Figure 3, the histopathological examination by hematoxylin and eosin (HE) staining of adipose tissue in HFD-fed and Ce6-only treated beagle dogs at 100× magnification revealed an increase in adipose size and a decrease in the number of adipocytes in each field. However, adipocyte hypertrophy and fat deposits were reduced by the Ce6-PDT treatment (both 10 and 15 min of LED light exposure) compared to the HFD-fed control.

### 2.4. Study of Ce6-PDT in C57BL6 Male Mice with HFD-Induced Obesity

**Figure 4:**
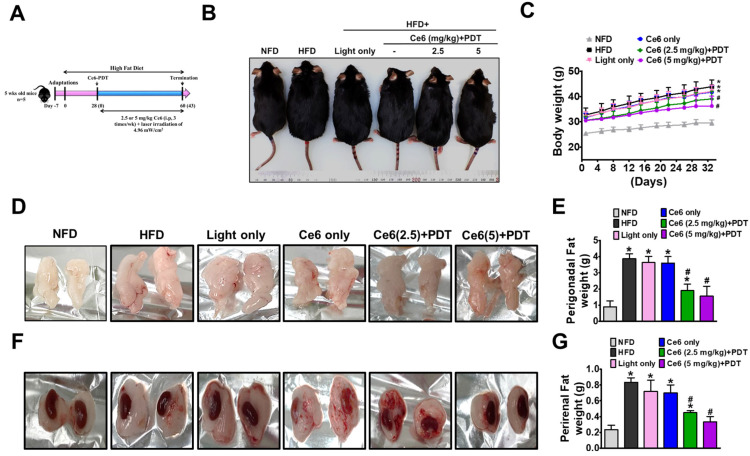
Ce6-PDT attenuates HFD-induced obesity in C57BL/6 male mice. (**A**) Experimental design; (**B**) Representative appearance of different groups of mice at 62 days after drug intervention; (**C**) Body weight changes (**D**); (**E**) Representative appearance of PG fat and its weight (**F**); (**G**) Representative appearance of PR fat and its weight. The data are presented as the mean ± SEM (*n* = 5). * *p* < 0.05 compared with the NFD group. ^#^ *p* < 0.05 compared with the HFD-only treated group.

To validate the effectiveness of Ce6-PDT in an in vivo animal model of obesity, male C57BL/6 mice were subjected to 60 days of HFD (60% fat) (Figure 4A). On the 28th day of HFD feeding, the mice in the HFD group exhibited a significantly greater body weight in comparison to their counterparts in the normal fat-diet (NFD) group. Then, the mice received vehicle or Ce6 (2.5 or 5 mg/kg), followed by exposure to 660 nm LED-light irradiation (4.96 mW/cm^2^) for a maximum of 45 days from the 28th to the 62nd day. The results showed that Ce6 (5 mg/kg) followed by a higher dose of PDT was significant in preventing HFD-induced obesity, as shown by the decline in the cumulative body weight of the mice. Ce6 (2.5 mg/kg)-PDT also showed a reduction in body weight but was lesser in comparison to Ce6 (5 mg/kg)-PDT (Figure 4B,C). Under HFD, the mass of fat pad was found to be elevated in both depots, i.e., perigonadal (PGF) (Figure 4D,E) and perirenal (PRF) (Figure 4F,G). Therefore, in mice given a medication dosage of either 2.5 mg/kg or 5 mg/kg, there was a notable decline in both the relative PGF and PRF along with body weight.

### 2.5. Ce6-PDT Improved Body Weight and Fat Accumulation in the HFD-Induced Obese Balb/c Mice

**Figure 5:**
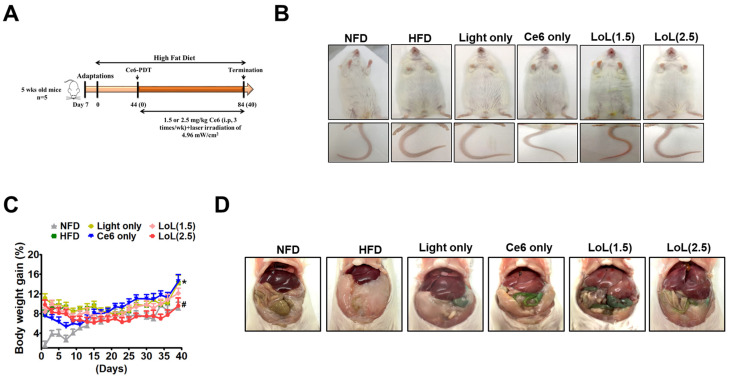
Ce6-PDT attenuates HFD-induced obesity in Balb/c mice: (**A**) Timeline for experiment; (**B**) mice from different groups; (**C**) body weight. The data are presented as the mean ± SEM (*n* = 5). * *p* < 0.05 compared with the NFD group. ^#^ *p* < 0.05 compared with the HFD-only treated group. (**D**) Distribution of white adipose tissue.

In order to evaluate the potential impact of the intraperitoneal administration of Ce6-PDT on the phenotype of mice following a high-fat diet, we tracked the weight gain of mice in an obese Balb/c mouse model (Figure 5A). Following 44 days of HFD feeding, Balb/c mice greatly accelerated the body weight by 30% (Figure 5B,C) compared to NFD. HFD feeding also increased the intra-abdominal adipose tissue more than in NFD (Figure 5D). Then, the mice received vehicle or Ce6 (1.5 or 2.5 mg/kg), followed by exposure to 660 nm LED-light irradiation (4.96 mW/cm^2^) for a maximum of 40 days from the 44th to the 84th day. Remarkably, the body weight was found to be decreased by treatment with Ce6 followed by LED-light irradiation (Figure 5C). Additionally, 40 days of Ce6-PDT treatment reduced fat abdominal density, as amply demonstrated by the anatomical investigations.

### 2.6. Ce6-PDT Suppressed Adipose Tissue Size and Distribution of White Adipose Tissue in Balb/c Mice with HFD-Induced Obesity

**Figure 6:**
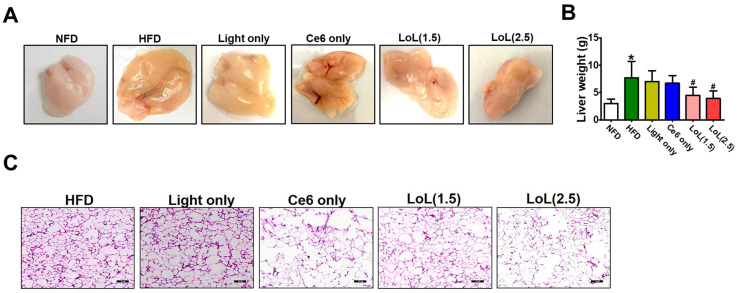
Balb/c mice displayed reduced adiposity when fed a HFD. (**A**) White adipose tissues; (**B**) fat pad weight. The data are presented as the mean ± SEM (*n* = 5). * *p* < 0.05 compared with the NFD group. ^#^ *p* < 0.05 compared with the HFD-only treated group. (**C**) Histopathological aspects of adipose tissue from mice with Masson’s trichrome staining. Scale: 50 μM.

Figure 6A illustrates the effects of HFD intervention and Ce6-PDT treatment on the composition of subcutaneous adipose tissue in mouse model 2. There were notable variations in the subcutaneous weight of adipose tissue between the groups. After 40 days, the mice fed HFD, light-only, or Ce6-only groups showed noticeably higher subcutaneous adipose fat mass in the unaided eye in comparison to the NFD groups. Ce6-PDT, both LoL (1.5) and LoL (2.5) treatments significantly decreased the fat mass compared with that of the HFD group (Figure 6B). This HFD-induced adipose tissue hypertrophy was significantly reduced by the Ce6-PDT in LoL (2.5) and LoL (1.5) treatment groups compared to HFD-only treated groups.

Obesity is defined by an increase in the adipocyte area rather than an increase in the adipocyte count [35]. Furthermore, the effect of Ce6-PDT treatment on adipocyte size was observed in subcutaneous fat via HNE staining. Figure 6C shows hypertrophic adipocytes in the HFD administration groups compared with the NFD-fed group. Ce6 (2.5) with PDT decreased adipocyte hypertrophy compared with that noted in HFD-fed groups. Adipocyte hypertrophy was decreased by Ce6 (1.5) with PDT, though less than by Ce6 (2.5) with PDT. Altogether, these results demonstrated that the Ce6-PDT treatment ameliorated HFD-induced obesity in Balb/c mice.

### 2.7. Ce6-PDT Improved Hepatic Steatosis in HFD-Induced Obese Balb/c Mice

**Figure 7:**
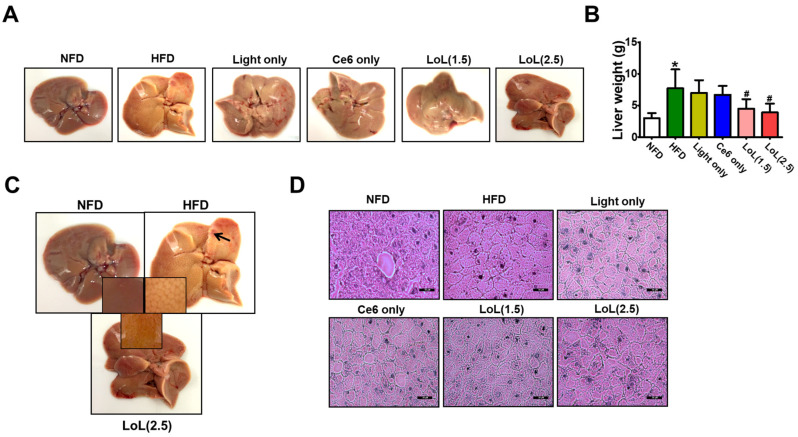
Ce6-PDT reduces hepatic steatosis in HFD-fed obese mice. (**A**) Liver morphology; (**B**) liver weight. The data are presented as the mean ± SEM *(n* = 5). * *p* < 0.05 compared with the NFD group. ^#^ *p* < 0.05 compared with the HFD-only treated group, and (**C**) comparative liver morphology in the NFD, HFD, and LoL (2.5) groups. A black arrow shows a discoloration of the liver in HFD mice. The highlighted image shows lipid droplets. (**D**) HE staining. Scale: 50 μM.

The impact of Ce6-PDT on the morphology of the liver tissue was assessed by means of a macroscopic examination. As shown in Figure 7A, the liver tissue of mice in the HFD group turned pale compared with that of the mice in the NFD group. In comparison to the HFD group, the morphological variation in the liver tissue was greatly inhibited by Ce6-PDT (LoL2.5). This was similar to the findings of previous studies [36,37]. Groups receiving Ce6-PDT also showed restored liver tissue morphology. Mice fed with the HFD diet develop fatty liver and hepatomegaly; therefore, we assessed whether Ce6-PDT could ameliorate the hepatic steatosis by examining the morphology and lipid accumulation in the liver sections. The liver and body weights of mice were found to increase by HFD feeding which were then reduced to the greatest extent by LoL (2.5) subsequently followed by LoL (1.5) (Figure 7B). Next, the liver morphology of the mice was examined using HE staining. As shown in Figure 7C, severe macrovesicular steatosis was detected in the livers of HFD-fed mice. More fat droplets, both microvesicular and macrovesicular, were observed in most hepatocytes of the HFD-fed mice. Liver cell volume was increased, fat was observed as macrovesicular droplets, and ballooning was observed in liver tissue. In comparison to the HFD group, hepatocellular steatosis and inflammation were reduced to varying degrees in the Ce6-PDT treatment groups. Among the Ce6 and low-PDT-treated groups, less steatosis was detected in comparison to the high-PDT-treated groups. This reduction was found to be more obvious in the LoL (2.5) group in comparison to the LoL (1.5) group (Figure 7D). Masson’s trichrome staining of liver sections (Figure S1) showed that Ce6-PDT treatment reduced liver damage in HFD-fed mice, which correlates with that of HE staining. Thus, Ce6-PDT ameliorates the development of HFD-induced hepatic steatosis and restores the hepatocyte pathology in the mice.

## 3. Discussion

Ce6 is a second-generation photosensitizer that is FDA-approved and meets the clinical requirements for PDT [38]. The rapid accumulation of Ce6 within a short period of time and its fast drug clearance are advantageous for preventing drug accumulation and its toxicity [39]. According to our previous study, Ce6 metabolic stability in dog liver tissue was 85.4% after 30 min of administration and was found to be more superior than drugs like verapamil, diazepam, etc. [40]. In the present study, we confirmed that the liver microsomal stability studies for Ce6 over 30 min revealed a >99% rate in mice (Catalog #: MSMCPL, Thermo Fischer Scientific, Waltham, MA, USA) and humans (Catalog #: M0317, Sigma-Aldrich, St. Louis, MO, USA), indicating that Ce6 is more stable in the liver than the positive control, Buspirone (Table S2). Ce6 in mouse and human plasma remained relatively low (4%) after 4 h of incubation at 37 °C (Table S3). Furthermore, to predict drug interactions, Ce6′s inhibitory ability for the cytochrome P450 (CYP) isoenzyme was examined, with Ce6-PDT showing the largest inhibitory effect on CYP1A2, CYP2C9, CYP2D6, and CYP3A4, and a slightly lesser effect on CYP2C19 (Table S3). We chose the Ce6 dosage (2.5 or 5 mg/kg) in the beagle dog, and mouse experiments based on the findings presented in our previous works [30]. In the clinic paper, a chlorin e6 derivative, Photolon (Belmedpreparaty, Minsk, Republic of Belarus), was delivered intravenously at a dose of 2.5 mg/kg at 3 h before illumination [41].

Although Ce6-PDT has been utilized in the past to treat canine cancers [42], this is the first time that we have shown in vivo evidence of Ce6-PDT’s ability to reduce ovariectomized HFD-induced beagle dogs. Mostly, male obese mice are selected over females, as clinical studies have found that obese females are at lower risk of developing non-alcoholic fatty liver disease (NAFLD) than males. In addition, male mice demonstrate an earlier body weight increase than female mice when administered an HFD diet [43]. However, according to the study, OVX female mice on a high-fat diet (HFD) mimic a similar level of visceral adiposity, a greater body fat percentage, and hepatic steatosis as HFD-fed male mice [12]. Thus, in our study, the anti-obesity benefits of Ce6-PDT were compared in both estrogen-deficient HFD-induced beagle dogs and HFD-induced male mice. In the present study, we also investigated Ce6-PDT’s impact on HFD-induced hepatic steatosis in Balb/c mice.

Our previous studies, as well as the present study, suggest that Ce6-PDT has lipid-lowering properties. Also, Ce6-PDT inhibited the adipocyte growth and lowered the lipid accumulation in 3T3 L1 cells by turning on AMPK [40,44]. An increase in fat mass and body weight is concerning for overall health. Our findings revealed that a 10% rise in body weight was observed by the end of the seventh week of HFD consumption in OVX female beagle dogs. This weight gain reflected the excess anabolic rate of triglyceride synthesis over catabolism. According to Gao et al., various wavelengths of light permeate tissues at differing depths due to light absorption and scattering by biological tissues. Short-wavelength ultraviolet-visible light (400–700 nm) has a lesser penetration depth than NIR light (700–1100 nm) up to the subcutaneous adipose tissue layer [45]. Nonetheless, Ce6 treatment followed by LED-light irradiation of 660 nm for either 10 or 15 min up to 35 days in HFD-induced OVX female beagle dogs, decreased their body weight gains. This demonstrates Ce6-PDT effects on fat accumulation, by enhancing body weight control. HFD-induced OVX dogs also resulted in excessive fat mass accumulation, or obesity, which results in metabolic disorders, such as dyslipidemia. In addition, both doses of light irradiation and Ce6 showed a decrease in total, subcutaneous, and visceral fat in HFD-induced OVX dogs.

Next, Ce6-PDT was also effective in suppressing cholesterol and LDL but did not show any effect on VLDL, HDL, or TG in HFD-treated OVX dogs. The current investigation shows that the OVX female dogs treated with Ce6-PDT (5 and 10 mg/kg) for 35 days had lower TC and LDL levels; however, no change in HDL levels was detected. Our earlier study in HFD-induced male mice likewise showed a decrease in lipid levels with Ce6-PDT treatment [40]. However, TG did not demonstrate the changes in 35 days. In another study, Ce6-PDT exerts anti-lipogenesis in sebocytes by lowering lipid accumulation, including cholesterol and triglycerides, via AMPK activation, indicating its potential to be employed for the treatment of acne vulgaris [46]. The variations in the species, dosage, method, and length of the intervention could be the cause of the differences between studies. Elevations in serum ALT and AST generally reflect hepatocyte injury [47]. In addition, a low AST/ALT ratio suggests NAFLD and NASH, increasing the risk of liver cirrhosis and hepatocellular carcinoma [48,49]. More importantly, our findings demonstrated that the Ce6-PDT treatment in HFD-induced beagle dogs showed a reduction in the elevation of AST and ALT levels compared with the AST and ALT groups after 35 days. In beagle dogs, Ce6-PDT treatment at doses of 10 and 15 mg/kg did not elicit hepatic toxicity (Table S1 and Figure S3). In addition, Ce6-PDT were also effective in reducing adiposity in HFD-induced OVX male beagle dogs.

In the case of C57BL/6 male mice, four weeks of HFD consumption increased the body weight by 10%. However, Ce6-PDT (2.5 and 5 mg/kg) also effectively reduced the body weight of HFD-fed male C57BL/6 mice. Further, Ce6-PDT significantly reduced the size of adipocytes in the C57BL/6 mouse model. Ce6-PDT (LoL5)-supplemented HFD-fed mice decreased the average size of visceral adipocytes by 60% over that of LoL2.5-supplemented HFD by 50% to that of a HFD alone. This indicates that Ce6-PDT is capable of decreasing the visceral adipose tissue weight in male C57BL/6 mice.

It has been demonstrated in clinical settings that consuming HFD plays a major role in the development of NAFLD [21,26]. In the current study, dietary composition was a major factor in the pathophysiology of obesity. Regular intake of the high-fat diet, which contained 60% calories from fat, caused significant modifications to the biochemistry of Balb/c mice’s blood, liver histology, and liver functioning in the second model. These results support earlier research showing that HFD triggers the development of several disease traits, including inflammation, oxidative stress, steatosis, and insulin resistance [21,50,51]. Since the onset of NAFLD is hepatic steatosis, developing drugs to relieve this condition may aid in the treatment of NAFLD. After the administration of HFD to the Balb/c male mouse model for 44 days, the body weight increased by 30%, and the present work effectively replicated the onset of HFD-induced hepatic steatosis. In obese states, intracellular lipids accumulate in non-adipose tissues, including the liver, heart, and muscle [52,53]. Human studies in obese NAFLD patients have demonstrated that nearly 60% of the triglycerides found in the liver arise from circulating free fatty acids, 60–80% of which come from adipose tissue lipolysis [54,55]. The morphological change in the liver tissue of Balb/c mice and liver weight were greatly inhibited by Ce6-PDT (LoL 2.5) as compared to the HFD group. Our histological analysis revealed that HFD mice exhibited large areas of diffuse hepatocyte steatosis with numerous round vacuoles of various sizes surrounding the nucleus. We observed the role of Ce6-PDT in decreasing hepatic weight and decelerating lipid accumulation in the liver of HFD-induced Balb/c mice and found that Ce6-PDT inhibit hepatic steatosis during obesity. This result is consistent with the earlier work in which endoscopic PDT-based duodenal mucosal resurfacing had an influence on lowering the hepatic lipid droplets during HE and Oil red O staining of fatty liver [56].

## 4. Materials and Methods

### 4.1. Study Dogs

Healthy female OVX beagle dogs, 11 months old, were purchased from the Orient Bio Jeongeup Center. After one week of acclimation, the beagles were subjected to a physical examination. All beagles were maintained in separate cages (length × width × height of 100 cm × 100 cm × 208 cm) in the animal facility on a 12 h light/dark cycle and 50–70% relative humidity with free access to water. Daily food consumption and physical activity were noted by the investigator. The experiment was approved with the approval of the Animal Experiment Ethics Committee of Notus Co., Ltd, Songdo, Incheon, Republic of Korea (NOTUS IACUC 1 8-KE-252).

### 4.2. Body Weight Increase Regimen

The normal diet (ND) consisted of 19% protein, 10% fat, and 71% carbohydrates, for a total of 3.6 kcal/g. Briefly, animals in the HFD group received a specially formulated diet that included 19.4% protein, 20.6% carbohydrate, and 60% fat, with total calories of 5.0 kcal/g. Every day, the dog received a weighed diet, and the leftover food was weighed the next day to compute the food intake ratio. After the dog’s body weight increased by 10% with the HFD after 49 days, we screened and randomly assigned them to four groups (4 dogs per group). Groups were as follows: HFD only; Ce6 only; Ce6 + 10 min-light treatment group; and Ce6 + 15 min-light treatment group. Ce6 (2.5 mg/kg) was prepared by dissolving in a vehicle (normal saline) and was orally administered for 35 days (49–84 days). The administration was carried out at intervals of three times per week. Thereafter, administration was performed at intervals of 5 times per week until the autopsy. Irradiation was conducted 3 times per week: 15 min for group 3 and 30 min for group 4 until day 49 from the start of the test substance administration. After 3 h of incubation, beagle dogs were made to wear an LED irradiation belt (DS41901) manufactured by Dongsung Pharmaceutical. Hair was removed from the abdominal and flank areas of the dog, and they were made to wear the LED irradiation belt that touched these body parts, resulting in irradiation through the skin. The belts provided a 660 nm light with a power of 7.3 mW/cm^2^ (10 min, 4.4 J/cm^2^) in Group 3 and 7.3 mW/cm^2^ (15 min, 6.6 J/cm^2^) in Group 4. The irradiation was performed on each dog five times per week until the autopsy. For all animals, the occurrence of dead animals, moribund animals, and abnormal animals was observed during the experimental period. General symptoms were observed once per day. The weight of each individual was measured using an electronic scale at intervals of 2 times/week or 3 times/week thereafter. All beagles were determined to be in good health by examining their physical health. Bi-weekly physical examinations were conducted by weighing animals and then using a 9-point body condition scoring (BCS) system. This grading system is divided into four categories: BCS scores of 1–3 indicate underweight; BCS scores of 4–5 indicate leanness; BCS scores of 6–7 indicate overweight; and BCS scores of 8–9 indicate obesity or extreme obesity, with the body weight curves determined [35]. The physical examination was performed by two investigators.

### 4.3. CT Scan

Abdominal radiography using a digital radiographic system was conducted twice, at 0 and 35 days after Ce6-PDT treatment. Under general anesthesia, the dogs were positioned on the CT table. Anesthesia was performed using Zoletil 50 (VIRBAC, Carros, France, 5 mg/kg) and xylazine (Rompun^®^, Bayer AG, Leverkusen, Germany, 2.5 mg/kg) after test substance administration (day 0) and autopsy day (day 35). CT scanning was performed using a 32-row multi-detector CT scanner (Alexion, Toshiba, Japan) to measure the amount of fat (mm^2^). The CT images included the diaphragm and continued caudally to the coxofemoral joint.

### 4.4. Blood Biochemical Test

A fresh blood sample was collected into BD vacutainer tubes (Carlsbad, CA, USA). Blood samples were taken twice, before the test substance administration (day 0) and on the day of autopsy (day 35). Blood samples were centrifuged at 3000 rpm for 15 min at 4 °C, and the samples were stored at −70 °C. All the serum parameters, including ALT, AST, cholesterol, TG, HDL, and LDL, were measured using an automated biochemical analyzer (Hitachi-720; Hitachi Medical, Tokyo, Japan).

### 4.5. Autopsy

After blood collection for clinical pathological examination, the animals were anesthetized and then euthanized by exsanguination by cutting the axillary artery and vein. All organs (including the gastrointestinal tract) of the body surface, subcutaneous, head, thoracic, and abdominal cavities were observed. Spleen, heart, lung, kidney, liver, and abdominal fat were removed and fixed in a 10% neutral buffered formalin solution.

### 4.6. Mice Model 1

Six-week-old C57BL/6 mice (n = 30) were obtained from Orient Bio Inc. (Seoul, Republic of Korea). Animals were housed at 20 ± 2 °C on a 12 h light/dark cycle and allowed free access to food and water. Following a week of acclimatization to the NFD, the diet was switched to an HFD with 60% fat. The HFD group was fed a high-fat diet (60% kcal fat; D12492, Research Diets, New Brunswick, NJ, USA; Table 1), and NFD with normal diet (10% kcal fat; 5L79, Research Diets, New Brunswick, NJ, USA; Table 1). Throughout the experiment, the bedding and water were changed once weekly, and the HFD was changed twice weekly to prevent fat oxidation. All the mouse experiments were reviewed and carried out with the approval of the Institutional Animal Care and Use Committee of the Dongsung Cancer Center under protocol IACUC #ds002106117-2. After the mice’s body weight increased by 10% with HFD after 49 days, we randomly assigned them to the following six experimental groups (5 mice/group): (1) NFD; (2) HFD; (3) HFD + light only; (4) HFD + Ce6 only; (5) HFD + Ce6 (2.5 mg/kg) with high PDT; and (6) HFD + Ce6 (5 mg/kg) with high PDT. HFD-fed mice were administered intraperitoneal injections with normal saline, while drug-treated groups received Ce6 (2.5 and 5 mg/kg), and after 3 h, PDT was applied with a fluence rate of 4.96 mW/cm^2^ for a duration of 10 min. Each mouse received PDT when kept in an LED mouse chamber with the required fluence rate, as detailed in our earlier research. The dose of delivered light energy (fluence rate, exposure time) was determined based on the published paper [40,45]. PDT was repeated after one day interval. Body weight was measured every two days a week. At the conclusion of the experiments (60 days), mice were sacrificed by cervical dislocation, and the liver and fat pads were excised and measured.

### 4.7. Mice Model 2

Specific pathogen-free male Balb/c (6-week-old) (n = 30) mice were purchased from Orient Bio Inc. (Seoul, Republic of Korea) and used after one week of acclimatization. Animals were housed at 20 ± 2 °C on a 12 h light/dark cycle and allowed free access to food and water. After a week, the diet was switched to an HFD with 60% fat. The normal diet (10% fat) and high-fat diet (60% fat) were purchased from LabAnimal Co. (Seoul, Republic of Korea). The study was approved by the Institutional Animal Care and Use Committee of the Dongsung Cancer Center under protocol IACUC #ds002106117-2. After the mice’s body weight increased by 30% with HFD in 44 days, healthy male mice were randomly assigned to the six following experimental groups (5 mice/group): (1) NFD; (2) HFD; (3) HFD + light only; (4) HFD + Ce6 only; (5) HFD + Ce6 (1.5 mg/kg) with low PDT; and (6) HFD + Ce6 (2.5 mg/kg) with low PDT. HFD-fed mice were given intraperitoneal (i.p.) injections with Ce6 (1.5 or 2.5 mg/kg), and after 3 h, PDT was applied with a fluence rate of 4.96 mW/cm^2^) for a duration of 5 min. PDT was repeated after every one day interval. Body weight was measured every two days a week. At the end of the experiments (84 days), mice were sacrificed by cervical dislocation, and the liver and fat pad were excised and assayed by histopathological examination.

### 4.8. Histopathological Examination

The fixed tissue underwent general tissue processing procedures such as trimming, dehydration, paraffin embedding, and sectioning to prepare specimens for histopathological examination, and the tissue cryosections were stained using HE or Masson’s Trichrome. The slides were studied using an optical microscope (Olympus BX53; Olympus Optical Co., Tokyo, Japan). Histopathological changes were observed.

### 4.9. Statistical Analysis

All the data are expressed as the mean ± SEM. Statistical analysis was performed using a one-way analysis of variance followed by Tukey’s post hoc test. *p* < 0.05 was considered statistically significant.

## 5. Conclusions

In conclusion, our study offers a method to lessen the burden of obesity and lower the danger of hepatic steatosis in HFD-induced beagle dogs and mice, respectively. We surmise that Ce6-PDT was effective in both HFD-induced OVX female dogs and HFD-induced male mice. Furthermore, Ce6-PDT ameliorates hepatic steatosis in HFD-induced obese mice. Overall, the therapeutic approach that our study suggested might be a promising approach for the development of therapy for obesity and obesity-related hepatic steatosis. However, investigations into the molecular mechanism of Ce6-PDT in hepatic steatosis should offer crucial insights for future clinical studies.

## Figures and Tables

**Table 1:** Composition of the high-fat diets and normal diets in mice model.

Component	High Fat Diet (60% kcal)	Normal Diet (10% kcal)
	g	g
Casein 80-mesh	200	200
L-Cystine	3	3
Corn Starch	0	315
Maltodextrin 10	125	35
Sucrose	68.8	350
Cellulose BW200	50	50
Soyabean Oil	25	25
Lard	245	20
Mineral Mix S10026	10	10
Dicalcium	13	13
Phosphate		
Calcium Carbonate	5.5	5.5
Potassium Citrate	16.5	16.5
Vitamin Mix 10001	10	10
Choline tartrate	2	2
FD and C dye	0.05	0.05

HFD (60% kcal fat) D12492, Research Diets, New Brunswick, NJ, USA; NFD (10% kcal fat) 5L79, Research Diets, New Brunswick, NJ, USA.

## Data Availability

The data are contained within the article.

## Supplementary Materials

The following supporting information can be downloaded at: https://www.mdpi.com/article/10.3390/ph17060729/s1, Figure S1: Histopathology of liver sections using Masson’s trichrome staining of liver sections; Figure S2: CT scan of abdominal fat tissue compartments of HFD treated with or without Ce6-PDT; Table S1: Histopathological examinations of vital organs to determine the cytotoxicity of Ce6-PDT; Figure S3: Histopathological examinations of vital organs; Table S2. Liver microsomal phase I stability (% of remaining after 30 min) of Ce6; Table S3. Plasma stability (half-life, h) of Ce6; Table S4. Inhibitory Activities of Ce6 in CYP450 (IC50 values).
