# Supplementary material for: Preventing High Fat Diet-Induced Obesity and Related Hepatic Steatosis by Chlorin e6-Mediated Photodynamic Therapy
Source: Pharmaceuticals (Basel). 2024 Jun 5;17(6):729. doi: 10.3390/ph17060729 (PMC11206563; doi:10.3390/ph17060729)
Supplement: Supplementary file 1 [file pharmaceuticals-17-00729-s001.zip › pharmaceuticals-3028165-supplementary.pdf]

Supplementary Information

Preventing High Fat Diet-induced obesity and related hepatic steatosis by Chlorin e6-mediated Photodynamic Therapy

Pallavi Gurung[1], Junmo Lim[2] and Yong-Wan Kim*

[1]Dongsung Cancer Center, Dongsung Pharmaceuticals Corporation, Daegu 41061, Republic of Korea

*Correspondence: thomas06@hanmail.net (YMK)

TABLE OF CONTENTS

S1. Histopathology of liver sections using **Masson's trichrome staining of liver sections**.

S2. CT scan of abdominal fat tissue compartments of HFD treated with or without Ce6-PDT.

Table S1. Histopathological examinations of vital organs to determine the cytotoxicity of Ce6-PDT.

S3. Histopathological examinations of vital organs

Table S2. Liver Microsomal Phase I Stability (% of remaining after 30 min) of Ce6

Table S3. Plasma Stability (half-life, h) of Ce6

Table S4. Inhibitory Activities of Ce6 in CYP450 (IC50 values)

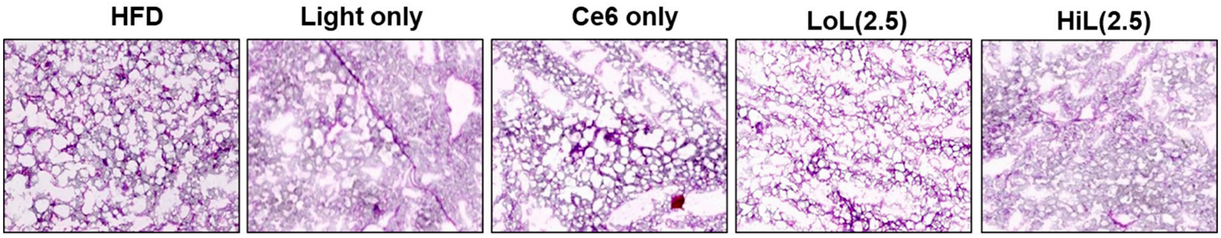

Figure S1. Histopathology of liver sections using Masson's trichrome staining of liver sections. Representative Masson's trichrome sections of steatosis in liver of HFD-fed mice exposed to light only, Ce6 only, LoL ((100x magnification)

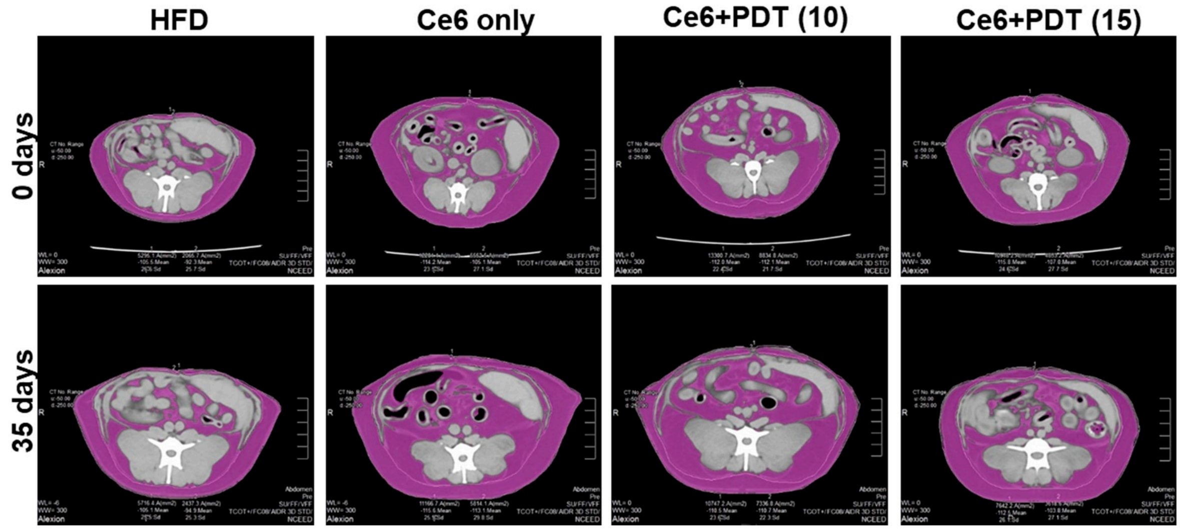

Figure S2. CT scan of abdominal fat tissue compartments of HFD treated with or without Ce6-PDT. Representative photographs MRI slices of abdominal fat in HFD, Ce6 only, Ce6+PDT (10), and Ce6+PDT (15) groups at 0 and 35 days.

Table S1. Histopathological examinations of different organs from the respective groups.

GROUPS	DOG	Histopathological Examination					
		Spleen	Heart	Lung	Kidney	Liver	Fat
HFD	1	NAD	NAD	NAD	Infiltration, inflammatory cells, severe pelvis	Inflammation, focal, mild	NAD
	2	NAD	NAD	Infiltration, inflammatory cells, subpleural	NAD	NAD	NAD
	3	NAD	NAD	Infiltration, inflammatory cells, perivascular	NAD	NAD	NAD
HFD+Ce6	4	NAD	NAD	Infiltration, inflammatory cells, perivascular	NAD	NAD	NAD
	5	Pigment, mild	NAD	Fibrosis, focal	Infiltration, inflammatory cells, mild, medullar	Inflammation, focal, mild	NAD
	6	NAD	NAD	NAD	NAD	Inflammation, focal, mild	NAD
HFD+Ce6 +PDT(10)	7	NAD	NAD	NAD	NAD	Inflammation, focal, mild	NAD
	8	Extramedullary haematopoiesis	NAD	Infiltration, inflammatory cells, subpleural	NAD	NAD	NAD
	9	NAD	NAD	NAD	NAD	NAD	NAD
HFD+Ce6 +PDT(15)	10	NAD	NAD	Infarction	NAD	NAD	NAD
	11	NAD	NAD	NAD	NAD	NAD	NAD
	12	NAD	NAD	Infiltration, inflammatory cells, perivascular	NAD	Inflammation, focal, mild	NAD

NAD: No Abnormality Detected

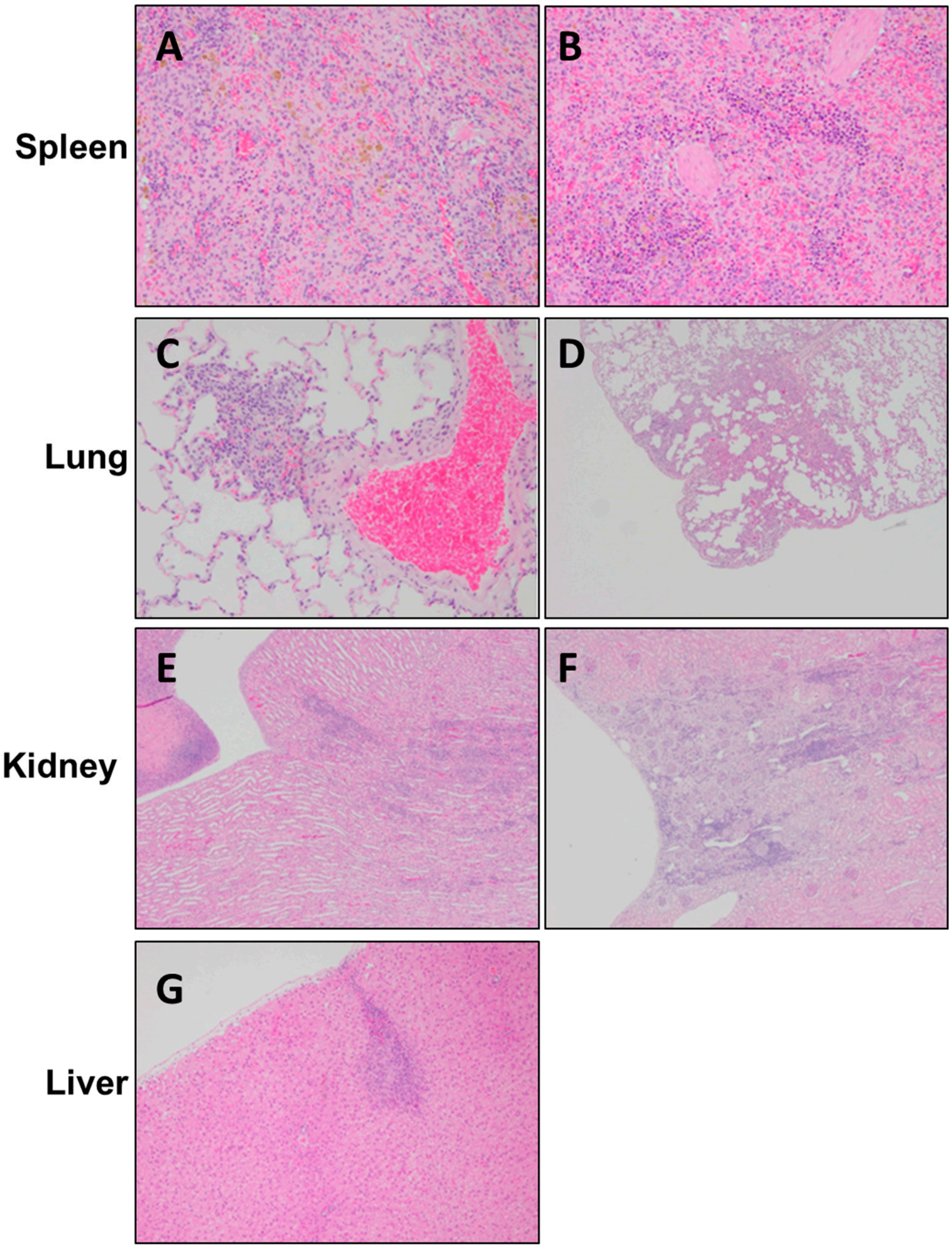

Figure S3. Histopathological examinations of vital organs. Histopathological examination was performed on the spleen, heart, lung, and liver. Most of the dogs showed no alterations with Ce6-PDT treatment. Some dogs showed histopathological changes. A: Spleen of Ce6 only group, Pigment X400; B: Spleen in Test article + laser irradiation 10 mins, Extramedullary hematopoiesis, X100, C: Lungs of Non-treated group showing Infiltration, inflammatory cells , X200 D: Lungs in Ce6 only group showing fibrosis , X40. E: Kidney of Non-treated group showing Infiltration, inflammatory cells , X40. F. Kidney of Test article + laser irradiation 15 mins showing Infarct, X40. G. Liver of Non-treated group showing inflammation at X100.

Table S2. Liver Microsomal Phase I Stability (% of remaining after 30 min) of Ce6

Compounds (1 μM)	Mouse (%)	Human (%)
Ce6	> 99	> 99
Buspirone	0.04 ± 0.01	4.28 ± 0.12

Table S3. Plasma Stability (half-life, h) of Ce6

Compounds (5 μM)	Mouse (%)	Human (%)
Ce6	> 4	> 4
Enalapril	24.5 ± 0.187	93.86 ± 0.301
Procaine	0.0198 ± 0.024	4.4 ± 0.5

Table S4. Inhibitory Activities of Ce6 in CYP450 (IC50 values)

Compounds	1A2	2C9	2C19	2D6	3A4
Ce6	>50	>50	23.7	>50	>50
Inhibitor (%)	99.6	95.5	83.2	97.7	99.1

1A2: α-naphthoflavone (10 μM), 2C9: sulfaphenazole (10 μM), 2C19: amitriptyline (100 μM), 2D6: Quinidine (10 μM), 3A4: Ketoconazole (10 μM)
